# Supervising Students During a Global Pandemic: Clinical Educators' Perceptions of a Student-Led Telerehabilitation Service During Covid-19

**DOI:** 10.5195/ijt.2022.6464

**Published:** 2022-06-03

**Authors:** Megan H Ross, Andrea Whitehead, Lauren Jeffery, Nicole Hartley, Trevor Russell

**Affiliations:** 1 Recover Injury Research Centre, The University of Queensland, Brisbane, Australia; 2 School of Health and Rehabilitation Sciences, The University of Queensland, Brisbane, Australia; 3 Business School, The University of Queensland, Brisbane, Australia

**Keywords:** Allied health, Clinical education, COVID-19, Telehealth, Telerehabilitation

## Abstract

**Scope:**

In March 2020, COVID-19 restrictions prompted services delivered by student-led clinics in the university sector to transition to telehealth. This provided a unique opportunity to explore the challenges and opportunities faced by clinical educators when supervising students to deliver telehealth.

**Methodology:**

Semi-structured interviews were conducted with allied health clinical educators who supervised students on clinical placement who were required to provide services via telehealth. Clinical educators across the disciplines of audiology, occupational therapy, physiotherapy, and speech pathology were asked to reflect on their experiences and perceptions of the rapid transition to a telehealth model for student clinical placements. A content analysis approach was used to analyse qualitative data.

**Conclusions:**

From the perspective of clinical educators, student-led telehealth services can effectively meet client needs while achieving student learning outcomes. This study highlights many opportunities for student learning via telehealth in the clinical education environment and the role of the clinical educator in the learning experience.

Lockdown restrictions and social distancing requirements in Australia during the COVID-19 pandemic in 2020 resulted in many healthcare consultations transitioning from in-person to a telehealth model of service delivery. Telehealth is a proven means of providing health care services to people who may otherwise be unable to access services due to barriers such as travel, finances, physical mobility, and access to suitable healthcare providers (Eikelboom et al., 2021; Horsley et al., 2019; Russell et al., 2011; Speyer et al., 2018). Prior to the COVID-19 pandemic, the utilisation of telehealth in Australia was limited (Wade et al., 2014). This included healthcare services that ran out of training facilities such as University clinics supervised by clinical educators (CEs) to support students on clinical placement.

The University of Queensland Health and Rehabilitation Clinics (UQHRCs) deliver allied health services across the disciplines of audiology (AUD), occupational therapy (OT), physiotherapy (PT), and speech pathology (SP). Within these clinics, students on clinical placement deliver in-person services to members of the public, under the supervision of discipline-specific CEs. Clinical educators are qualified allied health professionals, typically with several years clinical experience, who provide direct support, education and training to students while on clinical placement (Cantatore et al., 2016; Hewat et al., 2018). Clinical placement provides students with the opportunity to translate theoretical knowledge to clinical practice and to develop and demonstrate their clinical competence against discipline specific criteria under the guidance and supervision of CEs. Within the UQHRCs there is a dedicated Telehealth Clinic that provides services to the community in the disciplines of AUD, OT, and SP. Despite this, prior to the COVID-19 pandemic many of the CEs and students of the UQHRC had little or no experience in the supervision and delivery of clinical services via telehealth, with less than 12% of the total services delivered via telehealth in 2019.

With the restrictions created by COVID-19, many clinics, including the UQHRCs, underwent rapid transition to telehealth to ensure continuity of care and clinical placements. Since then, several studies have investigated the transition experience from the perspective of the client and health care professional (Filbay et al., 2021; Malliaras et al., 2021; Taylor et al., 2021). While these studies provide insight into the provision of telehealth services by experienced clinicians, the clinical education context is complex and unique, and as yet not thoroughly explored. Not only are CEs responsible for the care and safety of the client receiving services they are also responsible for mentoring and facilitating students to develop key clinical competencies. To meet emerging demand for future clinicians who can deliver efficient and effective telehealth services, understanding the nuances, challenges, and opportunities experienced by CEs in a student-led telehealth service is required. The aim of this study was to investigate allied health CE perspectives of a student-led telehealth service during COVID-19.

## METHODS

Data for this study were collected via in-depth semi-structured interviews to understand the CE perspective of clinical supervision of student-led telehealth services. Ethical approval was obtained from the University of Queensland Human Research Ethics Committee (Approval number: 2020000940). Informed consent was provided prior to interviews which were conducted between July and September 2020.

### PARTICIPANTS

CEs who supervised a student clinical placement between March and September 2020, that involved provision of healthcare services to clients via telehealth, were invited to participate in this study. CEs across the disciplines of AUD, OT, PT, and SP were invited to participate by clinic managers and participation was voluntary. The models of service provision and clinical education differed across disciplines. AUD and SP CEs and students were in their own respective homes (either in Australia or internationally), PT CEs and students were based at the UQHRC (i.e., co-located) and OT CEs and students were either co-located at the UQHRC or in their own respective homes. All clients were based at home, school, or their place of work.

### DATA COLLECTION

Semi-structured interviews (see Appendix) were conducted with CEs by an experienced qualitative researcher (MHR). The interview guide was developed to comprehensively garner CEs' perceptions of the supervision of students who rapidly transitioned to a telehealth service delivery model. Semi-structured interviews were selected as an appropriate method to collect in-depth data from CEs as the interviews allow probing for more information and clarification of responses (Barriball & While, 1994), and are well suited for exploring perceptions regarding complex issues.

Interviews were conducted and recorded via Zoom and were between 20 and 39 minutes in duration. Recordings were de-identified and saved according to university data management protocols, and transcribed verbatim.

### ANALYSIS

Thematic content analysis of interview transcripts was performed using NVivo software (NVivo qualitative data analysis software, 2012). The coding protocol was developed by one researcher (MHR) and refined at regular meetings with the research team members who have extensive experience across clinical education (AW, LJ), telehealth (AW, MR, TR), and qualitative research methodology (MHR, NH). Qualitative analysis occurred concurrently with data collection, which ceased once saturation was achieved. Double coding occurred with a minimum of 10% of the data to ensure consistency of emerging themes prior to finalising the coding protocol and analysis. Study rigour was guided by Tracy (2010) and all relevant markers and criteria of the Consolidated Criteria for Reporting of Qualitative Research (COREQ) (Tong et al., 2007) guidelines have been addressed.

## RESULTS

A total of 28 CEs supervised clinical placements that were delivered by telehealth at the UQHRCs during the study period; 17 (61%) responded and completed the interview. CEs were predominantly female (82%), between 27 and 54 years of age and the majority were from the SP (41%) and PT (35%) disciplines, with smaller numbers from AUD (18%) and OT (6%). Four (24%) CEs reported having previous experience with telehealth prior to COVID-19. Further detailed participant demographics are provided in Table 1.

**Table 1:** Participant Characteristics (n = 17)

Age, mean (SD) (range)	40.9 (8.2) (27 to 54)
Gender, n (%)	
*Female*	14 (82.4)
*Male*	3 (17.6)
Discipline, n (%)	
*Speech Pathology*	7 (41.2)
*Physiotherapy*	6 (35.3)
*Audiology*	3 (17.6)
*Occupational Therapy*	1 (5.9)
Clinic attended, n (%)	
*Speech Pathology*	7 (41.2)
*Physiotherapy - Neurological Aging & Balance Clinic*	4 (23.5)
*Physiotherapy - Musculoskeletal & Sports Injury Clinic*	2 (11.8)
*Audiology*	3 (17.6)
*Occupational Therapy*	1 (5.9)
Region, n (%)	
*Metropolitan*	15 (88.2)
*Regional*	2 (11.8)
Internet connection, n (%)	
*National Broadband Network (NBN)*	11 (64.7)
*ADSL / WiFi*	5 (29.4)
*Mobile (3G/4G)*	1 (5.9)
Previous experience with telehealth, n (%)	
*No*	13 (76.5)
*Yes*	4 (23.5)

**Figure 1:**
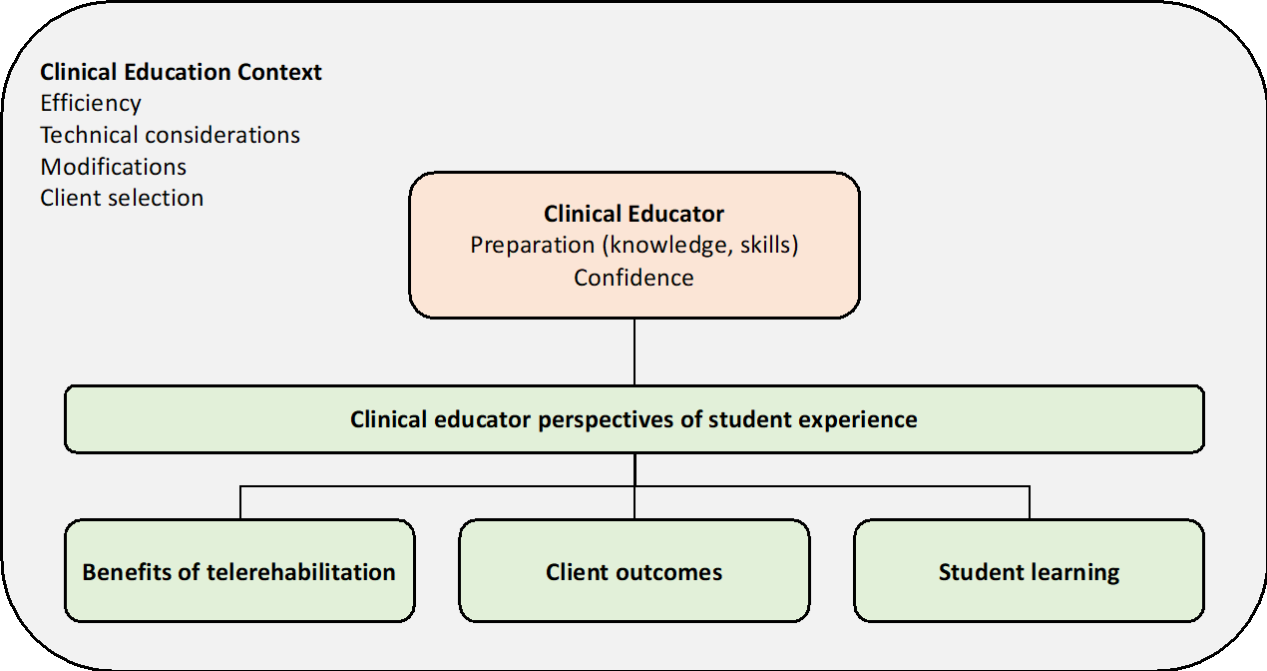
Schematic Representation of the Interplay between Key Themes and Subthemes

CEs described their perceptions and lived experiences of the rapid transition to a telehealth service delivery model in student-led clinics, and the key factors required for successful clinical supervision of telehealth placements. Three overarching, interrelated themes and nine subthemes emerged from the data (Figure 1). All themes and subthemes are described below, with individual participant quotes identified by a unique participant ID (e.g. CE004) assigned at the time of transcription.

### THEME 1: THE CLINICAL EDUCATOR’S ROLE IN TELEHEALTH CLINICAL PLACEMENTS

Two key factors underpinned the role of the CE in telehealth clinical placements: preparation (i.e., developing knowledge and skills) and confidence.

#### PREPARING CLINICAL EDUCATORS TO SUPERVISE STUDENTS TO DELIVER TELEHEALTH

Acknowledging the rapid transition to telehealth due to restrictions on in-person clinical supervision, CEs reflected on how they prepared themselves (i.e., developed their knowledge and skills) to supervise students delivering telehealth consultations. Three key strategies for enhancing knowledge and skills emerged. Clinical educators felt that *education, familiarisation* and *discussion with colleagues* facilitated preparedness to supervise student delivery of telehealth. Clinical educators noted that a combination of these elements tended to best facilitated preparation:

*“A lot of practice and research and watching of other speech pathologists or other clinicians in health providing services or how they have provided services via telehealth. The Speech Pathology Australia website had some fantastic resources as well that were developed in response to COVID…Then I think having a good play around with Zoom, learning more through the Zoom website, talking with colleagues, working with colleagues in clinic meetings to learn more about how to use Zoom for our service purposes.”* (CE004)

Access to *education* facilitated the transition to supervising student-led telehealth consultations. Many CEs described accessing resources, research, guides and modules, provided internally (i.e., independent learning packages, workshops and presentations) and externally (i.e., webinars and manuals provided by professional associations) to prepare themselves to supervise students. For example: “*…there was an APA [Australian Physiotherapy Association] webinar on it, and that was really helpful. I felt after that I understood more about what we were about to do, and I felt more confident then, going into it.”* (CE067)

Clinical educators discussed *familiarisation* with the software as both a barrier and facilitator for successful transition to supervising students to deliver telehealth consultations. Some reflected that “*greater familiarity with Zoom…and more training and experience in how to use the software would have been extremely helpful.”* (CE004) Others described the ways in which they upskilled and became more familiar with the software and how this was beneficial for them to then supervise students to deliver telehealth: “*I downloaded and practiced using Zoom prior. I asked my students to do the same”* (CE010).

Formal and informal *discussions with colleagues* facilitated preparedness to supervise telehealth consultations. Clinical educators discussed the benefits of having regular meetings, conversations with clinic managers, and the opportunity to ask questions of other CEs with more telehealth experience. These helped to allay fears, made them feel supported and feel “*confident that there was always that help.”* (CE063). For example: “*I could speak with the clinic manager, the student liaison staff and the clinic administrator…I had a fair bit of support from the other staff and we also had some meetings where we would catch up with other CEs via Zoom and talk over any issues.”* (CE016)

From their experience during the rapid transition, CEs had many suggestions and recommendations about how to further facilitate CE preparedness to supervise students to deliver telehealth consultations. These included professional development, training, inter-professional sessions, resources, and guides developed covering how to best deliver therapy via telehealth. For example:

*“I would say to watch some telehealth videos. I found watching a telehealth session, prior was super useful. I kept thinking back to that session and how my sessions would be similar or different. And that was a good model for me…All of the basic technical things about how to use the online platform that you're going to be using, whether it's Zoom or whatever other one. It'd be great to have links to resources that are great for assessment and therapy over tele. Links to research and evidence around tele and what works well and what doesn't, same as for students. A PDF to forward to students to ensure checklists to be completed prior to the clinic starting, those sorts of things.” (CE010)*

#### CONFIDENCE SUPERVISING STUDENTS TO DELIVER CARE VIA TELEHEALTH

While CEs felt confident in their experience and skills supervising students in-person, they expressed some concerns about translating and transferring their skills in student supervision to the telehealth model. For example: “*I was a little apprehensive, adding the layer of technology on having to supervise students, I had to think about something different than what they're doing clinically. I had to think about how best to deliver [care] online, and how I would be able to troubleshoot if something went wrong.”* (CE006). Confidence supervising students to deliver telehealth appeared to be discipline specific, in the context that disciplines utilising telehealth services prior to COVID-19 restrictions (e.g., SP) had greater confidence than those who did not (e.g., PT). Compared to those without prior experience with telehealth, CEs in SP had higher levels of personal and/or professional experience delivering telehealth, so were more confident in their ability to supervise students: “*I was feeling positive…with my own professional experience, offering services via telehealth…I had a good sense of what the process would look like.”* (CE004) Conversely, a CE in OT with no prior telehealth experience said: “*I wasn't feeling confident myself as a therapist to provide therapy using that sort of format, I was then unsure how I was going to support the students to do that.”* (CE045) Despite this lack of professional experience, CEs did feel they were able to draw on their skills from supervising students in-person and apply them to the telehealth supervision model. One CE described feeling “*confident I can use all the skills I've learnt doing a lot of face-to-face supervision”* (CE006) and another said, “*I feel like it is, kind of, the same as supervising in person.”* (CE016)

### THEME 2: STUDENT EXPERIENCE IN TELEHEALTH CLINICAL PLACEMENTS

Clinical educators' perceptions of the student experience were related to three key subthemes: client outcomes and improvement, benefits of telehealth and student learning.

#### CLIENT OUTCOMES AND IMPROVEMENT WITH STUDENT-LED TELEHEALTH SESSIONS

Overall, CEs felt they were able to provide clinical education to students sufficiently to achieve clinical outcomes for their clients. Perceptions of the level of client improvement with telehealth delivered by students (compared to in-person) were mixed and appeared to be discipline specific; SP CEs in particular had more positive perceptions of client improvement. Factors influencing clinical outcomes primarily related to common limitations of telehealth, and not the model of clinical education or the students' skills or proficiency (i.e., younger age, ability to engage via screen, or technological skills of the client) and reaching a ceiling effect for what was able to be done via telehealth (Table 2). For example:

**Table 2:** Key Themes and Subthemes Generated during Qualitative Analyses

Theme 1: The clinical educator's role in telerehabilitation clinical placements	
Subtheme 1: Preparing CEs to supervise students to deliver telerehabilitation (developing knowledge and skills)	
Education	“Yeah, so we were given some education through the university here, and [a telerehabilitaiton expert] gave us a small talk and were able to go through the independent learning packages provided by the university, and then also I probably found them, for my telehealth practice, I actually found the APA provided education was probably the most helpful, but that was nothing about clinical education provided in that. I think mostly it came up with stuff on the fly as well.” (CE022) “And also, I have had to read up on the evidence-based for telehealth service delivery with speech pathology, and from my readings and also participating in workshops or presentations around it, for speech pathology there's always more research needed. However, the research that was there was showing that it was no better but no worse than face-to-face delivery.” (CE060)
Familiarisation	“With developing skills, I think the most learning you do is when you actually try and implement the skills, so I think the first sessions that I had was where I probably learnt the most. I've been a clinical educator for a long time now, and I did have really rudimentary contingencies planned in case technology failed, and I sort of had that prepared, including pen and paper resources if I needed to use it if technology failed.” (CE060) “So we had done some practice ourselves, and had really upskilled ourselves in the week leading up to it, so I think it was more about the safety aspects of our population necessarily rather than the actual teleconsults and the technology side of things.” (CE062) “So we had those sessions, and they were generally about three hours with each manufacturer during that week. And then we wanted to have time to practise on each other as CEs and remote into each other and we all had our own little set of hearing aids and did all that practice at home. But it would have been good to have more time.” (CE063)
Discussion with colleagues	“We had a great team that was very supportive of each other and we asked each other lots of questions and talked through a lot leading up, so I think that allayed some of those fears in that way.” (CE062) “And when we started seeing real clients and having students see the real clients all via Zoom, we could easily just quickly text one of the other CEs and they would excuse themselves from their appointments and jump in and try and troubleshoot a situation. So in that way, it was actually really good because yeah, we felt confident that there was always that help.” (CE063)
Recommendations	“I guess it would have been good to be able to do an inter-professional kind of session and compare what other people were doing in their disciplines for certain troubleshooting situations. Even if we had done it for a couple of weeks, and then met and just not so much like an official training session, but just to touch base to see and communicate with each other about what was working, what was difficult. Because I had a very audiology focus, but maybe somebody else might have been able to contribute in a different way which we hadn't thought of, I guess.” (CE063) “I think sharing – sharing ideas on how to kind of carry out assessments via telehealth, how to – and different tools to use for therapy using that format. So, yeah, and also that technology piece, so having some training around using – not – I mean, Zoom technology is fairly easy but kind of being able to bring forward therapy sessions using technology basically. So that kind of assessment piece, I feel like I need some support in how to deliver formalised assessments on that effectively with Zoom. I suppose more about that training on different ways of carrying out different therapy using that format would be good, different computer-based sort of activities and how to sort of – you know, ways of carrying out assessments without being face-to-face. So, yeah, I guess more learning around that would be good.” (CE045) “So some of those things that I mentioned before I think in terms of adequately preparing the patient for the session, and some of those tips and tricks in terms of how to do things that will help you diagnostically and even to assess impairments. So just some of those ideas or things that have been tried before by experienced clinicians in the area. Maybe even going through like a whole – some case studies. Like, selecting certain case studies and just seeing how a physio experienced in tele manages the assessment that they actually did for someone with a suspected ACL or someone with acute lower back pain. And just seeing how they manage the assessment and management from a tele point of view. I reckon that would have been an interesting way actually of upskilling myself, rather than just general tips and tricks.” (CE011)
Subtheme 2: Confidence supervising students to deliver care via telerehabilitation	
	“You still use all of your other clinical skills but, for something new, I guess you're always going to have a steep learning curve at the beginning.” (CE010) “I was underconfident that we would be able to assist students to safely deliver effective sessions…I'm quite an experienced clinical educator, so I think that definitely helped. So I felt as well-equipped as I was likely to be, given I wasn't very experienced with telehealth. So my first experiences with telehealth were whilst supervising students. I think that the greatest assistance to me as a clinical educator in telehealth would be to have more experience in telehealth myself. And I think that doing it one-on-one with the client is really different from doing it while supervising a student. So I learned I probably only know how to supervise a session, rather than knowing how to lead one myself so it would be helpful if I'd done a bit more myself first. If we were to do a few more telehealth sessions of our own and I think, as a clinician, you reflect and think I could have done that better in this way. I'm going to try that next time and see if it works. Whereas we were trying to do that vicariously through students and, while we were able to say, that really didn't work, they weren't necessarily picking that up as quickly as what we would have. We would have gone on to the next idea to try within the session, so it was a slower learning process to do that through students than if I just jumped in and done a tonne myself and then go, oh, I know how to do telehealth more effectively and I can teach you to do that.” (CE012) “I feel very comfortable being a clinical educator in something face-to-face because I'm quite comfortable managing and training someone face-to-face. Whereas my very first clinical education experience doing a telehealth session was also my very first telehealth session that I'd ever done, so that was rather challenging.” (CE022)
Theme 2: Student experience in telerehabilitation clinical placements	
Subtheme 3: Client outcomes and improvement with student-led telerehabilitation sessions	
	“I felt that there was a bit of a ceiling effect with tele because there was limited progress in terms of, I didn't have the equipment I would need or I couldn't put my hands on or we couldn't progress an exercise for safety, or for we didn't have the right setup to do that. So I felt like we got people to as far as we could over a non-face-to-face contact and then it was, because they're making gains all the time, we weren't able to progress them in the end as quickly as we were in the beginning of the tele.” (CE062) “Checking home exercise programs in the clients setting, breaking down the barriers, them performing them at home and allowing them to do increase exercise therapy in their own setting. That was a really big thing that I thought would be quite helpful in the future.” (CE015)
Subtheme 4: Benefits of telerehabilitation for students in the context of student-led clinics	
	“I was really excited when we could do things like supervise students remotely. I think it was something that wouldn't have even been considered before, and so I thought this is great because it's another avenue we can go down, and it's technology we can utilise and offer student clinicians clinical placement experiences that they don't have to be in the same room with us to actually have those placements. So I think that was fantastic that we could change our services to provide that.” (CE060) “So they'll be learning all about the remote programming and yeah, about tele services I'm sure, because of yeah, what's happened. They'll need to, and the industry has changed as well. I mean in these few months they're going to need it, as graduates, to be up-to-date with what's happening in practice out there.” (CE063) “I thought only just that really I'm so glad that we did it, because I think the students that did the most of it, it really improved their skill set and it's great that they can use that skill set going forward into their careers when they graduate as another avenue for treatment. So I'm glad. Yeah, I'm really glad that we did it.” (CE067) “I think for UQ combine a real clinic and tele it will be the best. Say, as I said, in the clinic is really busy, but you've got hands-on, nothing can replace that. But, at the same time, if you've got other tutoring and other parts that you can go into online, is you actually more concentrate, so you more concentrate, you ask more questions, you get into more in-depth study and discussion. So, I think in the future that we can look at a combined of both scenario for the training, and that will be great for the student. Because all the time is tele is too much, but sometimes is tele is very flexible for the student and they are, as I said, their participation is great.” (CE021)
Subtheme 5: Student learning and improvement in telerehabilitation clinical placements	
Development of proficiency throughout placement	“…our students learnt a heap, not just in relation to therapy engagement but also how to do telehealth sessions as well. I can't think of anything really in particular that would make it better. Again, on the whole, there are those students who are really automatic in their ability to provide high quality care and other students who needed some support, but, yes, I think that that would have been the same whether it was face-to-face or tele. So I don't think that made any difference to the student's ability to demonstrate skill.” (CE009) “I think we challenged the students to think outside the square and by the end of the five weeks, the students that were performing to a good standard were able to, students that were only just passing or below adequacy definitely struggled more in a telehealth setting, as they would a lot of the time just fall back into repeating the session that they did before. I think part of it is, is that the students are struggling enough with neuro as it is when it's right in front of them, and them to be thrown the curveball of having to deliver this in a completely new medium that number one, their clinical educators are not much experienced in and they weren't expecting to deliver it in. Obviously, it depended on the quality of the student, as well. The ones that were going to do better, probably did better, anyway. The ones that were just barely passing or going to fail, struggled immensely in the telehealth setting.” (CE022)
Added complexity of telerehabilitation in a new clinical area	“I feel like they had that same nervousness around it and maybe it did add a little extra layer of being concerned.” (CE006) “I was concerned initially if I'm honest. Mainly because the students were struggling in the physical form, let alone then over tele, if that makes sense. So it was almost the first block of students and they were fresh in musculoskeletal alone, let alone anything else. And so I was wondering how that would transition.” (CE067) “…how to get up to speed with how to do the intervention and assessment and then also think about doing the same thing via telehealth. I think, it was probably just the student's ability to manage that new service delivery model, as an added layer on top of also providing the services that were new to them, as well, in terms of their experience.” (CE004) “I was wondering whether being via telerehabilitation…whether it would add an extra complication to the student changing their behaviour and picking a different task or making the glide less difficult, so changing how they administer it…[and]…whether they would be able to do that via Tele and troubleshoot the technical aspect of the exercise with the added layer of thinking about the technology and not being face-to-face.” (CE016)
Potential gaps in skill development	“…they don't necessarily always have the chance to feel and touch and move, which is very important in a neuro placement.” (CE062) “So they'll be learning all about the remote programming and yeah, about tele services I'm sure, because of yeah, what's happened. They'll need to, and the industry has changed as well. I mean in these few months they're going to need it, as graduates, to be up-to-date with what's happening in practice out there.” (CE063)
Theme 3: Telerehabilitation in the clinical education context	
Subtheme 6: Efficiency of telerehabilitation model for clinical education	
Perceived efficiency	“We would normally have at least three that we're supervising, but I can't with our current setup, can't see how we could supervise three. But if I could sit and look at three on the one screen and tap into or out of each one and have my audio do the same, I think that would be great.” (CE012)
Strategies implemented to improve efficiency	So there was just an extra added layer of complexity in terms of time. So I think overall the total amount achieved was less, but that was mitigated later in the weeks. Because once you would – you would invest time at setting things up really well in terms of treatment, so you'd set up an exercise really well, and then it would be really valuable, and they could do it as a home exercise program, they could do it in the next session without much set-up, because they knew exactly what they had to do. So it was an investment. (CE015)
Subtheme 7: Technical aspects of supervising student-led telerehabilitation sessions	
Technical skills of three parties	“I am technologically challenged for sure, but I feel the students, being techno natives, they actually helped quite a lot. They probably took to this faster and better than I did as a clinical educator. So I actually gained some support technologically anyway from the students.” (CE011)
Limitations and capabilities of the software platform	“It got to the point where to give the students enough experience, we were having to supervise multiple tele sessions sometimes and that was a bit tricky in terms of trying to figure out the best way to do two Zoom sessions. So we had to audio out of one and audio into the other, and I would keep both videos going so I could at least see what was going on but I wouldn't be able to hear the one audio. In terms of getting the numbers of clinical experiences up, I probably found the trickiest part of things in being able to supervise effectively over multiple tele sessions.” (CE062)
Subtheme 8: Modifications to student supervision to fit a telerehabilitation model of service delivery	
Modifications to therapy students delivered	“I had a good sense of how to provide services for fluency but, I think, it was then knowing how to do that through Zoom. That was different for me. I didn't feel prepared for that in those couple of initial weeks with the student clinic…I think, particularly for the paediatrics, you have to go in really prepared and be prepared to do things differently and have the availability of resources or ideas to manage that via telehealth.” (CE004) “My student did a very good job of making activities that worked in a tele format, so we didn't have anything that was not working. So when we work with children we play a lot of games, and obviously you can't have a child via tele roll a dice or hold a set of cards or any of those things. So the activities had to be modified to be used essentially on the computer and controlled always by the student, if that makes sense. The student did all of that herself.” (CE013) “I guess that surprised me that we could actually adapt to the format…the activities that the students were doing with the kids were suitable for that telehealth format. So a lot more was technology based, they were sending resources beforehand. So the expectations were quite clear in that way. They had to be very visual and so I think that, yeah, the students adapted and – yeah, in that way. So I guess – yeah, because – because of the style of the activities they did suit that telehealth format, so I guess it was easier for them to explain what they wanted the kids to do. We oulf have to adjust our goals to suit the telehealth format…we would adjust our goals and what we chose to work on to meet that context that they were in.” (CE045)
Modifications to supervision	“It was difficult when you wanted to give feedback if the interaction was going on with the student and the client. You could often in clinic whisper something in the student's ear without interrupting the whole session. Whereas via Zoom, I had to sit centre front to give that feedback. Once we learned that it was really effective to send chat just to the student and give them feedback as they were ready to receive it and modify the program via that chat that was helpful. I guess as educators, knowing that we could do that was good.” (CE012) “And then after that we have a lot more discussion say, okay is this - because after they seen the client they have different feelings, then we go into an in-depth discussion, okay what do you think, you know, or other things. Compared with the clinic, we have a lot more study with the case, but the clinic is, we just do more.” (CE021)
Subtheme 9: Selecting appropriate clients for student-led telerehabilitation services	
	“I think we actually handpicked the clients that we decided to use telehealth for, and we particularly did not pick clients that were relatively high-falls risk or really were attending the clinic for mostly balance exercises.” (CE022)

“*We had probably nearly half of the clients that grew and developed their skills, probably just as much as they would have in a face-to-face consultation. And a group of clients who certainly would have done better in face-to-face, just because of the needs that they have, their attention, their inability to really engage with the screen. So it wasn't so much about what the student was doing it was more about their inability to involve them in a bit more play and that sort of thing*.” (CE009)

Clinical educators also discussed supporting and working with students to develop goals and treatment aims that were appropriate for the mode of delivery, and how this facilitated their ability to meet client goals: “*Once we'd chosen our goals and felt that they were appropriate and achievable over tele, then we ensured they were met comprehensively…”* (CE010). Clinical educators felt client outcomes and improvement via telehealth were directly related to the benefits of increased access to therapy, being in the comfort of their own home (for assessing and exercising) and increased involvement/engagement with therapy delivered by students.

#### BENEFITS OF TELEHEALTH FOR STUDENTS IN THE CONTEXT OF STUDENT-LED CLINICS

Clinical educators described the additional benefits for students in terms of increased access (permitting remote supervision), learning to improve their communication skills (interpersonal skill development), ability to spend more time preparing, discussing and debriefing client cases and the additional skills added to their skill set upon graduation (Table 2). For example:

“…*the learning aspect, they actually learned quite a lot from it. It's also because we have more time so we're able to talk about the case or, if not, even introduce new things for them, that they wouldn't otherwise have been able to if they're in the clinic.”* (CE030)

While the ability to remotely supervise students delivering telehealth sessions was primarily considered positively, at times CEs were concerned about adequately supporting students who were in remote locations. For example, one CE said: “*I do have concerns for some of the remote students if they're having challenges…Just providing that support knowing they're remote, I just don't know if there's something else we need to be doing for those students…I just don't know if they feel a bit more isolated, particularly if they're underperforming…”* (CE060)

#### STUDENT LEARNING AND IMPROVEMENT IN TELEHEALTH CLINICAL PLACEMENTS

Perceptions of student learning and ability to achieve competency were related to: *development of proficiency throughout placement, added complexity in a new clinical area and potential gaps in skill development.* Clinical educators discussed the process of student learning and improvement over the course of the clinical placement block: “…*as a whole, they got better as the block went on.”* (CE067) Overall, CEs felt this occurred similarly on telehealth placements as it would have in-person. Specifically, those students who had difficulties over telehealth would likely have had difficulties in-person also: “*…there were some students who got their message across really adequately and their sessions were fantastic…then other students who…needed a bit more support…but my impression is that that would have happened whether it was face-to-face or tele anyway.”* (CE009)

Across all disciplines, CEs had concerns about students entering a new clinical area where they were developing and learning new skills, with the added complexity of simultaneously learning telehealth. Many discussed the added complexity of transferring new, novice clinical skills to fit a telehealth model of care and the additional stress and/or anxiety for students: “…*the added complexity…for students, I was very concerned that that would cause even more stress to deal with, not only general technical software issues that occur in audiology, but then adding Zoom to that.”* (CE063) Prior experience in the clinical area was discussed as both a barrier and facilitator for student ability to effectively deliver telehealth services. For example, one CE was concerned about how “*students with an intermediate level competency, and with no familiarity with telehealth at all prior to that, and also very new skills to the area of practice that the clinic related to…”* would be able to provide effective therapy and another felt that “…*success in the telehealth setting a lot of time relies on the experience of clinicians…”* (CE022).

While many CEs felt that telehealth placements enabled students to develop interpersonal, communication and time management skills equally or better than in-person placements, CEs discussed potential gaps in hands-on/practical skill development, and the effect this may have on future employability and the ability to provide an adequate standard of care. For example: “…*if things are largely tele-focused, how does that impact their skills? Particularly as a physio where you're doing a lot of hands-on assessments and feeling things and moving things and how that plays into your experience and ability, to then walk into a job that might require a lot of hands-on things.”* (CE062) The experience of a telehealth placement was also discussed as a benefit for student skill development and employability.

### THEME 3: TELEHEALTH IN THE CLINICAL EDUCATION CONTEXT

This theme encompasses CEs' experiences and perspectives of telehealth in the clinical education context related to four key areas: efficiency, technical considerations, modifications and client selection.

#### EFFICIENCY OF TELEHEALTH MODEL FOR CLINICAL EDUCATION

Concerns around the *efficiency* of CE supervised telehealth sessions delivered by allied health students were primarily related to time demands. Several CEs felt that supervising students to deliver telehealth services was not as efficient as supervising in-person, due to the inability to supervise more than one student at a time in the initial phases of the rapid transition to telehealth (compared to 3-4 in-person). For example: “*The biggest limitation is the amount of time compared with face-to-face- that it takes to supervise telehealth intervention…I'm really reluctant now to book in more than one [session] at time for an educator, whereas that's not very efficient use of educator resources.”* (CE012). These concerns were more evident in PT and AUD, where CEs had less experience supervising multiple clinical sessions at once. The second concern related to efficiency was loss of time during sessions because of poor set-up and preparation (on the student or client's end) resulting in less actual ‘therapy’ time for clients. Strategies to mitigate this were implemented and included preparing clients and students in advance (encouraging planning sheets) to address issues such as space, equipment required, and preempting technology/software concerns. For example:

“*Early on, we found clients were wasting a lot of time trying to find…whatever equipment they needed for the consult. So we found that made things better if we made them aware of what they will need for the physio sessions, so they are ready in advance.*” (CE011)

Throughout the process, CEs also implemented changes to improve the efficiency of providing student feedback in-session, which included utilising the Zoom chat function and attending the telehealth session as a participant. For example:

“*We learned that it was really effective to send chat just to the student and give them feedback as they were ready to receive it and modify the program via that chat, that was helpful…it was [also] more effective once we were able to get on headphones and on our separate screen and Zoom into the meeting as a participant in the meeting and then give feedback that way.”* (CE015)

#### TECHNICAL ASPECTS OF SUPERVISING STUDENT-LED TELEHEALTH SESSIONS

Technical aspects that were unique to clinical supervision of student-led telehealth sessions were related to the *technical skills of participants* (CE, student and client) and *limitations and capabilities of the software platform*. While CEs did describe some common technical issues (e.g., poor connectivity, issues with audio/video and hardware/device concerns), these were infrequent and easily resolved. Rather than being concerned about the student's own technical abilities, CEs were concerned about the students' ability to guide clients through the technology, as well as the clients' own technical skills. One CE described having “…*some concerns about guiding the client through the technology…also teaching the student to guide the client through the technology.”* (CE012) From a clinical supervision perspective, CEs discussed some limitations with the software platform including initial difficulties supervising multiple students at a time (as described in Theme 3) which were resolved. “…*We were talking about how I could be in multiple Zoom meetings at once and then, the next thing you know, we're able to be in multiple Zoom meetings at once.”* (CE067) As discussed in Theme 3, other capabilities of the software afforded effective provision of feedback to students and facilitated clinical supervision:

“*…utilising the chat function a bit more readily, rather than actually jumping in and intervening too much in the telehealth sessions. Because if you can model things for students without necessarily completely taking over the session…utilising the chat function was quite useful because students could choose when to take on that advice or feedback. They didn't have to read it straight away, that was probably, in terms of the technology side, the biggest thing that I learnt, and I think that we as a CE team learnt to use.”* (CE022)

#### MODIFICATIONS TO STUDENT SUPERVISION TO FIT A TELEHEALTH MODEL OF SERVICE DELIVERY

All CEs described making modifications to clinical services to fit the telehealth model in two distinct ways. First, CEs described facilitating students to *modify their approach for performing assessment and delivering therapy* via telehealth. Some CEs who had not supervised or delivered telehealth sessions themselves, primarily in PT, were concerned about how to facilitate students to modify and provide effective services via telehealth (related to Theme 2): “…*I didn't really understand exactly what assessments were possible through telehealth, so that made it very difficult when planning assessment, to set the students up to succeed…”* (CE022) Overall, most CEs felt students were able to modify assessments, activities and resources that worked well in a telehealth format (Table 2). In physiotherapy, CEs at times felt students had difficulties ‘*thinking outside the box’* and beyond the standardised way of assessment and treatment. For example:

“*You've really got to use your creativity and I feel like maybe some of them were too black and white… [as a student] you feel you have to do it by the rule book, which is not necessarily the case. You can use any outcome measure over tele as long as you use it again the next time…I think maybe we were too prescriptive and black and white in the boundaries of what they thought they could do over tele*.” (CE062)

Second, CEs also made *modifications to their approach to student supervision*, primarily related to providing student feedback. Compared to in-person supervision, some CEs felt it was more anxiety-inducing to provide student feedback mid-session via the video/audio function in Zoom, and opted to use the chat function instead. Providing mid-session feedback this way allowed CEs to give students greater ability to attempt things independently and delayed CEs intervening (unless unsafe):

“*…in terms of choosing to intervene at a better time, it gave me the perspective of allowing students to have a go over tele. Because we would sit in a room separate from the student and Zoom in…we didn't need to physically be in the same room, we could use the Chat function to prompt them. If it wasn't obviously a huge safety risk, we could say, “Have you seen this?” Or, “Maybe try this,” and then they got to actually implement it rather than us maybe jumping in as quickly as we might have in a gym environment. But because we're solely for students here, it's obviously very easy to try and change things or modify things for them to give them the best learning experience.”* (CE062)

Other CEs felt that the telehealth model of supervision availed them more time to provide student feedback than in-person models of clinical education (Table 2).

#### SELECTING APPROPRIATE CLIENTS FOR STUDENT-LED TELEHEALTH SERVICES

Clinical educators discussed how the ability to select appropriate clients for students to manage via telehealth facilitated success for both the student and client. In neurological physiotherapy and audiology in particular, CEs felt this was essential. Client selection was influenced by factors related to client safety (level of supervision/assistance required), availability of/familiarity with technology, and level of mobility, cognition and/or hearing loss. For example:

“*We were very selective in the clients we picked. I was confident that the clients that were selected would be able to have a certain level of care via telehealth. Clients were familiar to the clinic, and they were independently mobile to some degree, with or without an aide, and they were low falls risk*.” (CE015)

In SP, CEs felt that telehealth was appropriate, and achievable for students to deliver services to a wide range of their typical clients.

## DISCUSSION

To our knowledge, this is the first study to explore CE perspectives of supervising students delivering care via telehealth during COVID-19. Overall, CEs perceptions and lived experiences of the implementation of a telehealth service were positive. Nine key themes emerged from the data and highlight the many opportunities for telehealth services delivered in allied health student-led clinics in the future and provide strategies to navigate potential challenges encountered through the implementation process.

The need for clinical education opportunities and continued service provision during the COVID-19 pandemic prompted the transition to telehealth across all disciplines in the UQHRCs. Clinical educators transitioned to supervising an effective clinical service that both facilitated access to therapy and continuity of care for clients and maintained a clinical education case load for allied health students on placements. While the nature of the rapid introduction of restrictions meant that opportunities for preparation and training in the use of telehealth were limited, CEs identified and discussed the strategies they utilised to upskill themselves, including peer-mentoring, self-directed learning and accessing professional development resources. The importance of CEs who are confident and capable of delivering effective telehealth has been identified as an important factor for student learning experience (Pit et al., 2021). While participants were experienced CEs, findings indicate that telehealth specific upskilling (including both technical skills and modifications to student supervision to fit a telehealth model of service delivery) is required for CE confidence, and to facilitate positive student and client outcomes. In this study, CEs in SP found the transition to telehealth easier than colleagues in the other disciplines. This most likely reflects the greater uptake of telehealth in the profession both at UQHRC and more broadly (Campbell & Goldstein, 2022). Future implementation strategies should include development of context specific training resources for CEs which address the unique aspects of the clinical education environment (e.g., methods of providing student feedback).

Acknowledging and responding to the unique requirements of the clinical education context will provide opportunities for successful implementation of telehealth delivered services in student-led clinics. Clinical educators found that supervising students to deliver telehealth was inherently different to standard clinician delivered services. On clinical placements, CEs are the gatekeepers to ensuring client safety, and facilitating a positive, and safe, student learning experience. In this study, CEs described strategies employed to overcome challenges that arose, and the opportunities to enhance student learning and the mode of clinical supervision in the future. While CEs acknowledged some challenges in the context of safety of clients, particularly in PT (e.g., due to lack of physical proximity for balance or unfamiliar tasks, or with young, unsupervised clients in OT/SP) similar to previous research, this also provided an opportunity for students to develop their awareness of, and to mitigate risks (Bridgman et al., 2022; Hong et al., 2020). Other challenges CEs described have been previously reported in the literature, including the lack of ‘hands-on’ experience (Malliaras et al., 2021), difficulties with clinical equipment and device fitting (Eikelboom et al., 2021) and managing clinical (diagnostic) uncertainty (Dawson et al., 2020). These concerns were more pronounced for CEs in AUD and PT. While challenging, CEs described how the experience provided opportunities for students to develop and refine fundamental clinical and interpersonal skills, including managing clinical uncertainty (Gheihman et al., 2020).

Delivering telehealth services in student-led clinics can provide a valuable learning experience for student clinicians. All CEs in this study, but particularly those in SP, felt that learning objectives were able to be maintained, and positive client outcomes were able to be achieved through the telehealth mode of delivery. The model provided an opportunity for students to develop interpersonal, communication, planning and problem solving (Dawson et al., 2020) and clinical reasoning skills. This is consistent with a study showing that students felt telehealth afforded them a greater opportunity to develop their clinical reasoning and communication skills (Dawson et al., 2020). The benefits also extend to equipping the future workforce with clinicians who are confident and competent with telehealth.

Students had the opportunity to access unique experiences in a model of care that is predicted to play an increasingly significant role in healthcare in the future (Waseh & Dicker, 2019). As training and awareness among students (and new graduates) has been identified as a contributing factor to clinician reluctance to adopt telehealth (Silverman, 2003), increasing student experience, familiarity and proficiency with telehealth is vital. Embedding telehealth into undergraduate coursework (Pit et al., 2021) is essential for adequately preparing future allied health clinicians to deliver effective telehealth services to clients. This is particularly important given the lessons learnt from a global pandemic and the necessary innovation required for ongoing health service delivery. Introduction of telehealth in allied health programmes may support greater uptake and acceptance of telehealth as a viable service delivery model in professions such as PT, who found the transition more challenging.

The transition to telehealth also provided UQHRCs the opportunity to explore the flexibility and adaptability of clinical placements to serve clinical education needs. Allied health programmes find it increasingly challenging to provide and secure suitable in-person clinical placement opportunities that meet accreditation requirements (in duration and type of clinical experience required) (Held et al., 2019). Increased clinical placement opportunities afforded via the implementation of telehealth supervised placements may provide a potential solution to meet this demand. The implementation of telehealth allowed students to continue with planned clinical placements, and the experience and knowledge gained through the initial lockdown period of 2020 has been expanded on in 2021. Intermittent COVID-19 related restrictions over the previous 18 months in Australia have continued to impact on clinical placements, and UQHRCs have been able to adapt to telehealth easily and flexibly, to ensure continuity of clinical care, student learning and progression to graduation.

The findings from this study have wide-ranging implications for CEs, clinical placement providers and students. While university-based student-led clinics are not necessarily faced with the same challenges as private practice or hospital-based settings (e.g., client complexity, case load demands, equipment access), the overarching themes are broadly applicable to all clinical education contexts. Clinical placement co-ordinators cannot assume that that CEs will be confident or proficient in the delivery of services via telehealth and need specific training, and systems and processes in place for success (Table 3). Clinical education facilities have the opportunity to select clients who students will be capable of managing via telehealth, and to utilise a hybrid approach to telehealth services (combination of in-person and telehealth delivered sessions). Given the benefits for developing students' skills in foundational clinical areas of communication, reasoning and planning, and the transference of skills to other clinical settings and to meet the emerging demand for efficient telehealth services, tertiary education institutes should consider the feasibility of incorporating a component of telehealth clinical education for all students.

**Table 3:** Key Practical Considerations for Supervising Student-led Telerehabilitation Clinical Placements

Table of Key Practical Considerations	
Suggestion	Explanation
Training	
Telerehabilitation “champions”	Key people in the team who have experience and expertise in telerehabilitation. For example, mentors who can provide training, troubleshooting advice and peer guidance.
Clinical educator training specific to telerehabilitation	Clinical educators require telerehabilitation specific training (in addition to standard clinical education professional development). Training should include: content related to the clinical provision of telerehabilitation (for CEs who do not have personal experience providing telerehabilitation services) including simulations telerehabilitation software/platform training and modifications to the clinical education model including student assessment tools (e.g., COMPASS, APP, SPEF-R)
Student coursework	Embedding telerehabilitation into undergraduate coursework should be considered to facilitate student familiarity and confidence prior to clinical placements. This should include learning modules and simulations.
**Pre-session Planning**	
Student preparation	In addition to familiarisation with the technology and platform, encourage students to complete a planning sheet to facilitate consideration of: environmental set-up for client equipment required for student and client additional resources required modifications that may need to be made to assessment and treatment techniques
Client preparation	Encourage students to: develop and share resources with family/client in advance complete a test call with the client on the day prior to ensure adequate technology, appropriate set-up, location and to discuss home equipment to have available for the session
Client selection	Clinical educators should select clients who are appropriate for the level of student learning. Consider client factors such as safety, comprehension, mobility, technical skills.
**In-session**	
Joining telerehabilitation sessions as a ‘participant’	Clinical educators may benefit from joining the telerehabilitation session on a separate device as a participant and observe the session from the client perspective.
Breakout rooms	Breakout rooms may be useful to supervise multiple concurrent telerehabilitation sessions delivered by individual students with their clients.
Chat function	Utilising the chat function may be beneficial for providing supervision / input mid-session without interrupting the telerehabilitation session.
**Student Learning Considerations**	
Placement selection and order	Where possible, students may benefit from having some hands-on/in-person experience in the clinical area before introducing telerehabilitation.
Placement model	The hybrid model of placement (a combination of in-person and telerehabilitation sessions) may be beneficial for students and clinical competency, so that skill development can occur across both in-person and telerehabilitation modes.

There are some methodological considerations relevant to the findings of this study. The results reflect the perceptions of CEs at one University in Brisbane, Australia and predominantly represent the views of CEs in the SP and PT disciplines. The sample size, and sampling rate (61%) is also a consideration; while all CEs were invited to participate, there is a possibility that those with strong opinions were more likely to respond. Findings are also specific to student-led clinics, which should be considered as a strength of this study. This study is the first to address the additional complexities of implementing telehealth in clinical supervision models.

## CONCLUSION

Clinical educators can effectively supervise students to deliver telehealth services that meet client needs, and student learning outcomes. To facilitate implementation and the student learning experience, CEs require telehealth specific training and upskilling to ensure confidence and proficiency. The telehealth model affords students many opportunities to develop clinical, interpersonal, and professional skills, and should be considered a viable clinical placement option for all allied health students to meet future demand for telehealth services.

## LIST OF ABBREVIATIONS

CE: clinical educator
UQHRC: University of Queensland Health and Rehabilitation Clinic
AUD: audiology
OT: occupational therapy
PT: physiotherapy
SP: speech pathology
APA: Australian Physiotherapy Association

## DECLARATIONS

### AVAILABILITY OF DATA AND MATERIALS

The datasets used and/or analysed during the current study are available from the corresponding author on reasonable request.

## APPENDIX

### Interview Guide

1. Prior to supervising your first student delivering telerehabilitation:
  - How did you feel the consultations would go?
  - Did you feel well prepared to supervise a student to deliver telerehabilitation?
2. Thinking back to your students' telerehabilitation sessions. In general…
  - From a technical perspective: What worked and what didn't work?
  - From a treatment perspective – what worked / what didn't work?
  - Did you have any concerns about any aspect of the consultation? What were these?
  - Do you feel the consultations helped the clients? If so, do you think they improved more / less / same as if they had an in-person consultation? Why do you think this?
  - Do you have any suggestions to make the service better?
3. Do you feel the students were able to deliver the same level/quality of care as you would have in an in-person consultation? Why / why not?
4. Do you feel like you needed additional training in order to have provided a better supervision service to student practicing telerehabilitation? If so, what elements of training do you think would have been helpful?
5. Now that you have supervised students delivering a telerehabilitation service, how would you rate your skills to supervise students delivering care via telerehabilitation?
6. Now that you have supervised students delivering a telerehabilitation service, how would you rate your knowledge to supervise students delivering care via telerehabilitation?
7. Now that you have supervised students delivering a telerehabilitation service, how would you rate your confidence to supervise students delivering care via telerehabilitation?
8. Did you feel adequately supported in supervising students who were delivering telerehabilitation care?
9. Do you think the students were adequately prepared to deliver telerehabilitation services? Why / why not? What else do you think they needed?
